# Male-specific phosphorylated SR proteins in adult flies of the Mediterranean Fruitfly *Ceratitis capitata*

**DOI:** 10.1186/1471-2156-15-S2-S6

**Published:** 2014-12-01

**Authors:** Giuseppe Saccone, Christos Louis, Hongyou Zhang, Valeria Petrella, Manuela Di Natale, Maria Perri, Marco Salvemini

**Affiliations:** 1 Department of Biology, University of Naples Federico II, 80134, Naples, Italy; 2 Institute of Molecular Biology and Biotechnology, Foundation of Research and Technology-Hellas, GR - 70013 Heraklion, Crete, Greece; 3 State Key Laboratory of Agricultural Microbiology and Institute of Urban and Horticultural Pests, College of Plant Science and Technology, Huazhong Agricultural University, Wuhan 430070, People's Republic of China

## Abstract

Alternative splicing is a widely used mechanism of gene regulation in sex determination pathways of Insects. In species from orders as distant as Diptera, Hymenoptera and Coleoptera, female differentiation relies on the activities of conserved splicing regulators, TRA and TRA-2, promoting female-specific expression of the global effector *doublesex *(*dsx*). Less understood is to what extent post-translational modifications of splicing regulators plays a role in this pathway. In *Drosophila melanogaster *phosphorylation of TRA, TRA-2 and the general RBP1 factor by the LAMMER kinase *doa *(*darkener of apricot*) is required for proper female sex determination. To explore whether this is a general feature of the pathway we examined sex-specific differences in phosphorylation levels of SR splicing factors in the dipteran species *D. melanogaster, Ceratitis capitata *(Medfly) and *Musca domestica *(Housefly). We found a distinct and reproducible pattern of male-specific phosphorylation on protein extracts enriched for SR proteins in *C. capitata *suggesting that differential phosphorylation may also contribute to the regulation of sex-specific splicing in the Medfly.

## Introduction

Alternative splicing (AS) can be regulated in tissue-specific and/or stage-specific manners and can be responsive to signaling cues [1]. Regulation of alternative splicing is achieved through the interaction of *trans*-acting splicing regulator complexes with the global splicing machinery. Gene-specific AS is regulated by differential expression, degradation, phosphorylation and intracellular localization of splicing factors [2]. Each splicing factor can have antagonistic effects on the alternative splicing outputs on few specific genes or on coordinated group of genes [3,4].

Serine/arginine-rich (SR) proteins are splicing factors involved in the regulation of several developmental processes, including sex determination in Insects, which share a RRM-type RNA binding domain and are rich in serine/arginine residues [5-9]. Phosphorylation of the serine/arginine repeats provokes a structural change that alters the functional state of these splicing regulators, thereby affecting developmental choices [10-13]. Protein phosphorylation plays an essential role in the regulation of sex-specific splicing in *Drosophila *[10]. The LAMMER kinase DOA is responsible for phosphorylating the SR protein RBP1 and most likely other two splicing factors: TRA-2 (SR protein) and the female-specific TRA (serine arginine rich, lacking the RMM domain), which promote female-specific splicing of *doublesex*. DOA phosphorylates also a second SR major protein, DX16, which is the *Drosophila *ortholog of the human SFRS7 9G8 SR protein [14].

In the Mediterranean fruit fly *C. capitata *and in the housefly *M. domestica*, the mechanism of sex determination of *Drosophila *is partially conserved [15-19]. In these species, TRA and TRA-2, which are related to SR proteins, function as splicing regulators of *doublesex *(*dsx*) and *fruitless *(*fru*) pre-mRNAs, promoting their female-specific expression and, ultimately, female sexual differentiation [16,20-25]. Orthologs of the *Drosophila tra *gene in different insect orders appear to be ON/OFF master switch genes for sex determination and are female-specifically expressed through alternative splicing [26]. Furthermore, except for *Drosophila, tra *is also able to regulate itself, promoting female-specific splicing of its own pre-mRNA to maintain continuous expression of TRA activity [27,28]. It is still unknown, though, how the male-specific splicing of *tra*, which leads to truncated, non-functional TRA isoforms, is regulated by the male determining factors in *C. capitata *and *M. domestica *[25,29,30]. Furthermore it is not yet clear if and how many downstream genes promoting sexual differentiation are also being controlled, during development and in different tissues, by sex-specific alternative splicing in *Drosophila *and other insect species [31-35].

To investigate if sex-specific SR phosphorylation takes place in adult dipteran flies, we used a commercially available mouse monoclonal antibody (mAb104), which surprisingly recognizes a highly conserved phosphoepitope shared by several major SR proteins (6-9) in metazoans, including vertebrates [36,37]. The SR family comprises at least 7 distinct major proteins in *Drosophila *and 9 in *Homo sapiens*; they all share the mAb104-phosphoepitope, which is also conserved in plants [38-41]. Interestingly, this antibody cross-reacts also with *Drosophila *TRA and TRA-2 baculovirus-expressed recombinant proteins in HeLa extracts, possibly owing to their phosphorylated SR domains promoted by human cell kinases [5]. We searched for sex-specific differences in phosphorylation of major SR splicing factors at adult stages of flies in the three distantly related dipteran species mentioned above (*D. melanogaster, C. capitata *and *M. domestica*), which are phylogenetically distant 130 million of years (mya), with *C. capitata *and *Drosophila *more closely related (120 mya).

## Results and discussion

**Figure 1:**
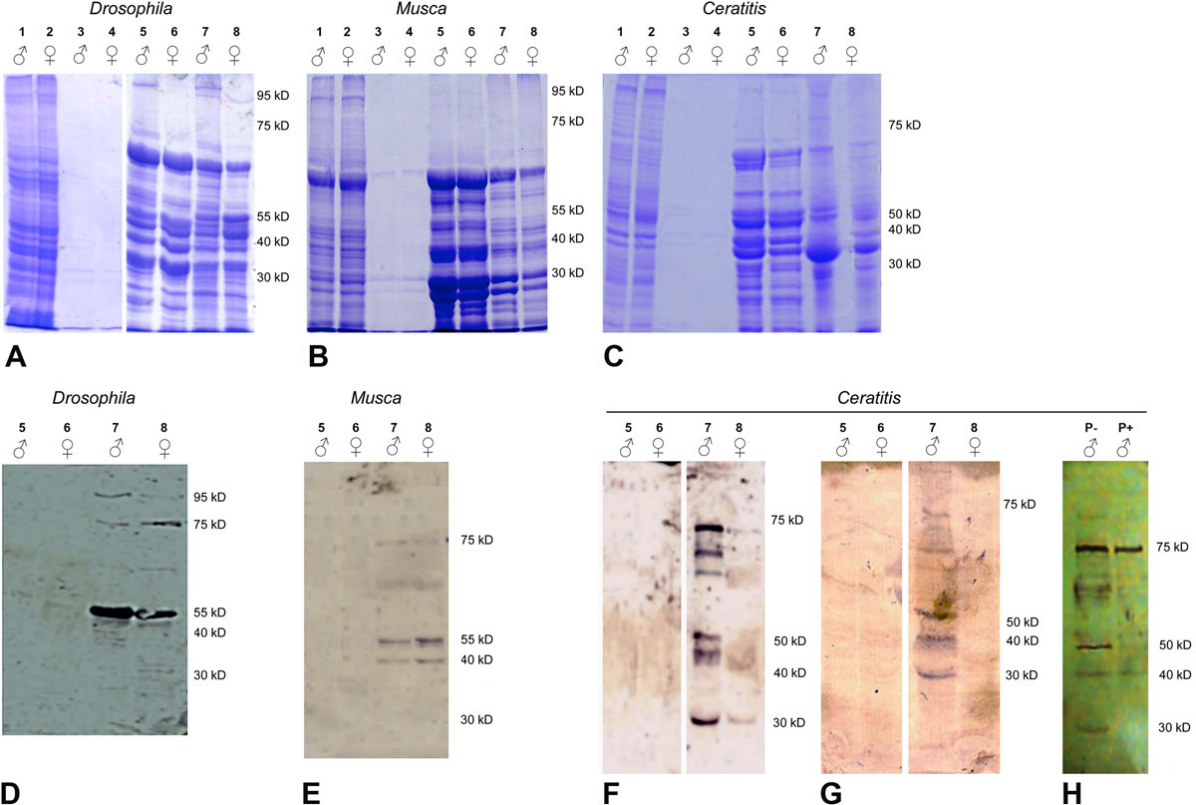
**SDS-PAGE electropherograms (A-B-C) and immunoblots (D-E-F-G-H) produced with protein extracts from adult males and females of *Drosophila, M. domestica *and *C. capitata***. 1) total lysate of males; 2) total lysate of females; 3) 90% ammonium sulfate precipitation supernatant of males; 4) 90% ammonium sulfate precipitation supernatant of females; 5) Mg^++ ^supernatant of males; 6) Mg^++ ^supernatant of females; 7) Mg^++ ^pellets of males; 8) Mg^++ ^pellets of females; P-) Mg^++ ^pellets of *C. capitata *males without phosphatase treatment; P+) Mg^++ ^pellets of *C. capitata *males with phosphatase treatment. After transfer to nitrocellulose, blots were incubated with either mAb104 or no primary antibody (as a control; data not shown).

To search for sex-specific differences in the phosphorylation of major SR splicing factors in *D. melanogaster, C. capitata *and *M. domestica*, proteins were extracted from male and female adult tissues. We enriched for SR proteins following a two-step protocol as described in [41]. In Figure 1 whole staining of SR protein enriched extracts as well as immunoblots with the mAb104 antibody are depicted. In all samples we recovered a pattern of enriched SR proteins similar in both males and females (Figure 1A, B and C). Following quantitation of the supernatant proteins and of the soluble proteins after magnesium precipitation from the last purification step, SDS-PAGE separations were performed in parallel duplicates, with one stained with Coomassie Blue (to control for equal loading) and a second one blotted onto a filter. The transfer of proteins extracted from each sex was controlled by reversible Ponceau staining, which is a validated method to assess both equal gel loading and protein transfer [42,43] (Additional file 1 - Figure S1). No sex-specific differences were evident. The filters were used for immunostaing and, on the contrary, showed some sex-specific differences. In *Drosophila *adult flies, the mAb104 antibody detects 6-8 polypeptides of molecular weights similar to those previously observed in *Drosophila *Kc cell line [37]. However, the relative amounts appear different in males and females (odd numbers: males; even numbers: females). For instance, the 95 kDa signal is significantly stronger in males, while the 75 kDa signal is more prominent in females (Figure 1D, lanes 7-8). In *M. domestica *four major phophorylated SR proteins were detected with comparable levels in both sexes (Figure 1E, Figure lanes 7-8). In two immunoblots with samples isolated from two different biological replicates of adult *C. capitata *flies, we observed that phosphorylated polypeptides were highly enriched only in males (Figure 1, lanes 7 of blots in F and G). Two of the six major SR antigens detected (~75 kDa and ~30 kDa) can also be seen at much lower levels in the female samples in one of the 2 blots (Figure 1, blot in F, lanes 8). As only 0.04% of cell proteins are SR proteins [39] and the mAb104 can detect them only by Mg*^++ ^*enrichment, we propose that the male-specific *C. capitata *SR antigens correspond most likely to phosphorylated SR proteins expressed in most of the fly tissues rather then small tissues (such as for example the male germ line).

When samples were treated with a phosphatase prior to immunoblotting, four of the six bands detected in male extracts disappeared (Figure 1H, lane P+). The ones that persisted even after phosphatase treatment could also be detected in the female sample (~75 kDa and ~30 kDa not shown). These signals probably result from mAb104 epitopes which were resistant to phosphatase treatment. This data suggests that the male-specific pattern observed in the *C. capitata *mAb104 immunoblots is due to male-specific phosphorylation for at least some of the SR proteins.

The absent (or strongly reduced) mAb104 signal in females could be due to absence of protein phosphorylation or to a lower expression of these SR proteins and corresponding genes. As a first step to investigate this question, we investigated by digital differential expression analysis (DGE) in adult *C. capitata *males and females using available RNA-seq data sets. The male and female reads were combined together and *de novo *assembled using Trinity software. We obtained 188,625 assembled transcripts with an N50 value of 1693 bp (data not shown). Using this data set six *C. capitata *SR orthologous proteins were identified by TBLASTN analysis and found to be highly conserved with the respect of the six *Drosophila *SR polypeptides (CcB52, CcRBP1, CcSC35, CcSRP54, CcX16 and CcASF/SF2, showing respectively 88%, 95%, 98%, 92%, 74% and 84% amino acid identity when compared to *Drosophila*). Male and female reads were then used separately to compute gene expression levels in both sexes (Additional file 2 - Table S1). We used the male-specific *beta-tubulin *and the female-specific *fst *genes of *Ceratitis *as a preliminary DGE controls and found that the former is exclusively expressed in the male sex while the latter gene is only expressed in females. Although we have no replicates for this DGE analysis and, hence, we cannot measure the sex bias, this first control data is consistent with what was expected, suggesting that we could answer to the question whether SR-encoding mRNAs are present or not in females. When we used the six Ceratitis SR genes for DGE, no one showed absence of expression in females. We have choosen CcB52 for further analysis, we performed an RT-PCR, using two pair of primers and we got cDNA fragments in both sexes of adult flies. These data suggests that the observed male-specific phosphorylation patterns are likely due to increased phosphorylation rather than to higher transcription in males or reduced transcription in females.

In metazoans SR phosphorylation is mainly controlled by a serine-threonine kinase specific for the SR domain, which is therefore named SR protein-specific kinase 1 or SRPK1 [45]. In addition, the Cdc2-like kinase (CLK) family has also been implicated in SR protein phosphorylation events [46]. We therefore investigated whether SR-specific kinases are expressed only in *C. capitata *males. We identified two putative *Ceratitis *SRPK1-related kinases and expression was observed in the two sexes by DGE analysis. A similar result was obtained for the *Drosophila *CLK kinase *darkener of apricot *(*doa*) ortholog in *C. capitata *which seems to produce mRNAs also in the adult females. The *Drosophila doa *gene expresses female-specific isoforms which may be involved in sex-specific SR phosphorylation events [47,48]. Further investigations would be needed to clarify if there are also alternative isoforms for some of these genes in *C. capitata*, which could have been differentially expressed in the two sexes.

## Conclusions

In conclusion, we show that in two distantly related dipteran species, such as *Drosophila *and the housefly, the phosphorylation pattern of SR proteins detected by the mAb104 in adult flies seems to be non sex-specific. In contrast in *C. capitata *adult flies the SR phosphorylation mAb104 pattern of six proteins is almost exclusively male-specific or strongly enriched in this sex. The DGE data showed that in *C. capitata *the six SR encoding genes are transcribed and most likely translated also in the female adult flies. Based on our study we can only speculate that 1) the 6 Ceratitis SR proteins are transcribed and expressed in both sexes to perform basal alternative splicing regulations, related to their deep evolutionary metazoan conservation and 2) that the male-specific mAb104-detected SR phosphorylated proteins could have additional functions, possibly related to maleness, and to the control of sex-specific or sex-biased gene splicing [49,50]. As Ceratitis, Musca and Drosophila show a conservation of *tra *and *dsx *sex-specific splicing regulation, but only *Ceratitis *show male-specific SR phoshorylated proteins, we propose that these splicing factors are involved in regulatory events either upstream or downstream to the tra>dsx regulatory module.

We think that the observation of male-specific phosphorylated SR proteins in *Ceratitis *male flies is a preliminary necessary premise to provoke interest and to start further *in silico *and functional analysis also in other Tephritidae. Future experiments including SR proteins extraction from dissected *XY* fly body parts and from sexed embryos/ larvae will help to understand where and when the male-specific SR phosphorylation pattern is present, although we presently think that these SR antigens are present in most of the tissues of the adult male fly. Immunoprecipitation and sequencing will help to find further confirmation and information about identity of the SR antigens and related potential isoforms.

## Materials and methods

### Immunoblotting

3-4 gr of flies were used for each protein sample and the final Mg^++ ^pellet (4th purification step) was resuspended in solution and entirely loaded in one gel lane. During the purification procedure an aliquot (10 microliters) from each of three different steps was saved for gel electrophoresis analyses. Tissues were ground to a fine powder in liquid nitrogen, using a mortar and pestle, and transferred to 30 ml of the isolation buffer (65 mM KCl, 15 mM NaCl, 10 mM HEPES at pH 7.6, 10 mM Na_2_EDTA, 5 mM DTT, 5 mM potassium fluoride (KF), 5 mM p-glycerophosphate, 0.2 mM PMSF, and 2 g/ml of aprotinin). The samples were sonicated and centrifuged to eliminate debris; from the supernatant a first aliquot of total proteins was saved from each sample for gel analysis (Figure 1, A-B-C, lanes 1-2). The supernatant was used for a 65% (2 hr of stirring at 4°C, and centrifugation at 8000g for 20 min) and then a 90% ammonium sulfate precipitations (2 hr of stirring at 4°C and centrifugation at 10,000 g at 4°C for 1 hr). A second aliquot (proteins from the sup 90%) from the supernatant of the centrifuged 90% ammonium sulfate precipitation was saved for analyses (Figure 1, A-B-C, lanes 3-4). The pellets were resuspended in 200 microl of dialysis buffer (65 mM Kcl, 15 mM NaCl, 10 mM HEPES at pH 7.6, 1 mM Na_2_EDTA, 2 mM DTT, 5 mM KF, 5 mM p-glycerophosphate, and 0.2 mM PMSF), dialyzed against three changes of 300 ml of dialysis buffer over the course of 9 hr. The dialysate was recovered and stored at -80°C. The dialysate samples were thawed and centrifuged for 15 min at 13,000g. Supernatants were transferred to clean tubes, and MgCl_2_ was added to 20 mM. After a 1-hr incubation on ice, tubes were centrifuged at 13,000g for 30 min. After removal of the supernatants, from which samples were saved for gel analyses (Figure 1, A-B-C, lanes 5-6), the pellets were washed with 200 microliters of 20 mM MgCl_2 _dialysis buffer and resuspended in 20 micro1iters of 5% glycerol buffer D [41]. Sample buffer was added to the various samples obtained from the purification procedure and the proteins were separated by SDS-10% PAGE (Figure 1, A-B-C, lanes 7-8). Similarly to the observations of Zahler *et al*. [41], the complexity of the protein samples throughout the 4 purification steps, was gradually reduced from step 1 to step 4, leading approximately to Mg^++ ^pellets containing 1-2 dozens prominent polypeptides visible by blue Coomassie stain in the 3 species. However, the observed grade of purification of the SR proteins (lanes 7-8) is lower than expected, being the electrophoretic pattern more complex if compared to the one described by Roth et al. and by Zahler et al. [36,41]. This could be due to the fact that in our study entire animals were used as starting samples, which are expected to be more complex compared to cell lines or dissected tissues in which it has been previously estimated that at least 0.04% of cell proteins are SR proteins [41]. However, most of the visible polypeptides in lanes 7 and 8 in the blue comassie are not detected by the mAb104 in the corresponding immunoblots (D-E-F-G).

### *De novo *assembly and digital expression analysis of medfly adult sexed transcriptomes

Illumina RNA-seq raw reads were downloaded from NCBI SRA homepage (0-6 days old adult Medfly males: SRX272878; 0-6 days old adult Medfly females: SRX272877). The male and female paired end (PE) reads were combined together and *de novo *assembled using Trinity software (release 2012-10-05) [51] on the ALAN Server at the Department of Biology of the University of Naples Federico II (24 cores, 192 GB of memory). Trinity was run on the PE reads with the fixed default k-mer size of 25, minimum contig length of 200, maximum length expected between fragment pairs of 500, 22 CPUs, and a butterfly HeapSpace of 20 GB. We obtained 188,625 assembled transcripts with an N50 value of 1693 bp. This adult data set was searched by TBLASTN analysis using as queries the *D. melanogaster *protein sequences of six SR genes (FlyBase Acc. Numbers: B52= FBpp0300515, RBP1= FBpp0081754, SC35= FBpp0088838, SRP45= FBpp0079471, X16= FBpp0078996, SF2= FBpp0082724) and of ten Serine/Threonine kinase genes (SRPK= FBpp0303478; srpk79D= FBpp0297551; Doa= FBpp0289029) and the *C. capitata *orthologs were identified (showing in *C. capitata *respectively 96%, 94%, 97%, 97%, 99%, 81%, 88%, 84%, 71% and 91% of aa identity; in case of multiple isoforms the longest one was selected as representative). Male and female reads were then used separately to compute gene expression levels in both sexes, using FPKM metric, by the RSEM software [52], the Bowtie aligner [53] and the edgeR software [54], as implemented in the Trinity software package with default parameters. The *C. capitata *male-specifically expressed *beta tubulin *gene (*Ccbeta2t*) [55] the female-specifically expressed *fst *gene [56] were used to assess the lack of contaminations of one sex with the other in the assembled adult transcriptomes.

## Competing interests

The authors declare that they have no competing interests.

## Authors' contributions

GS, CL, MS wrote the paper. MD, MP, VP performed molecular experiments. MS performed bioinformatic and DGE analyses. CL and HZ supported the research with materials and technical ideas. MS. GS conceived the concept of the study, supervised and coordinated experiments. Read and approved the final manuscript: GS, CL, ZH, VP, MD, MP, MS,

## Supplementary Material

Additional file 1 **Figure S1 Reversible red-ponceau staining of filters, used subsequently for mAb104 immuno-reactions**. Figure 1: Ponceau staining of blots from SDS page electrophoresis of SR protein extracts of adult males and females of *Drosophila *(D), *M. domestica *(E) and *C. capitata (*F and G). 5) Mg^++ ^supernatant of males; 6) Mg^++ ^supernatant of females; 7) Mg^++ ^pellets of males; 8) Mg^++ ^pellets of females; P-) Mg^++ ^pellets of *C. capitata *males without phosphatase treatment; P+) Mg^++ ^pellets of *C. capitata *males with phosphatase treatment. Click here for file

Additional file 2 **Table S1 Transcriptome analysis of SR-protein and Serine/Threonine kinase encoding genes in adult *C***. *capitata*. Expression levels for Medfly males (XY) and females (XX) are reported using FPKM metric (Fragments Per Kilobase Of Exon Per Million Fragments Mapped). LogFC= log_2 _of fold change values; LogCPM= log_2 _of counts-per-million of mapped reads; FDR= false discovery rate. Click here for file
