# Supplementary figures and images for: Male-specific phosphorylated SR proteins in adult flies of the Mediterranean Fruitfly Ceratitis capitata
Source: BMC Genet. 2014 Dec 1;15(Suppl 2):S6. doi: 10.1186/1471-2156-15-S2-S6 (PMC4255826; doi:10.1186/1471-2156-15-S2-S6)

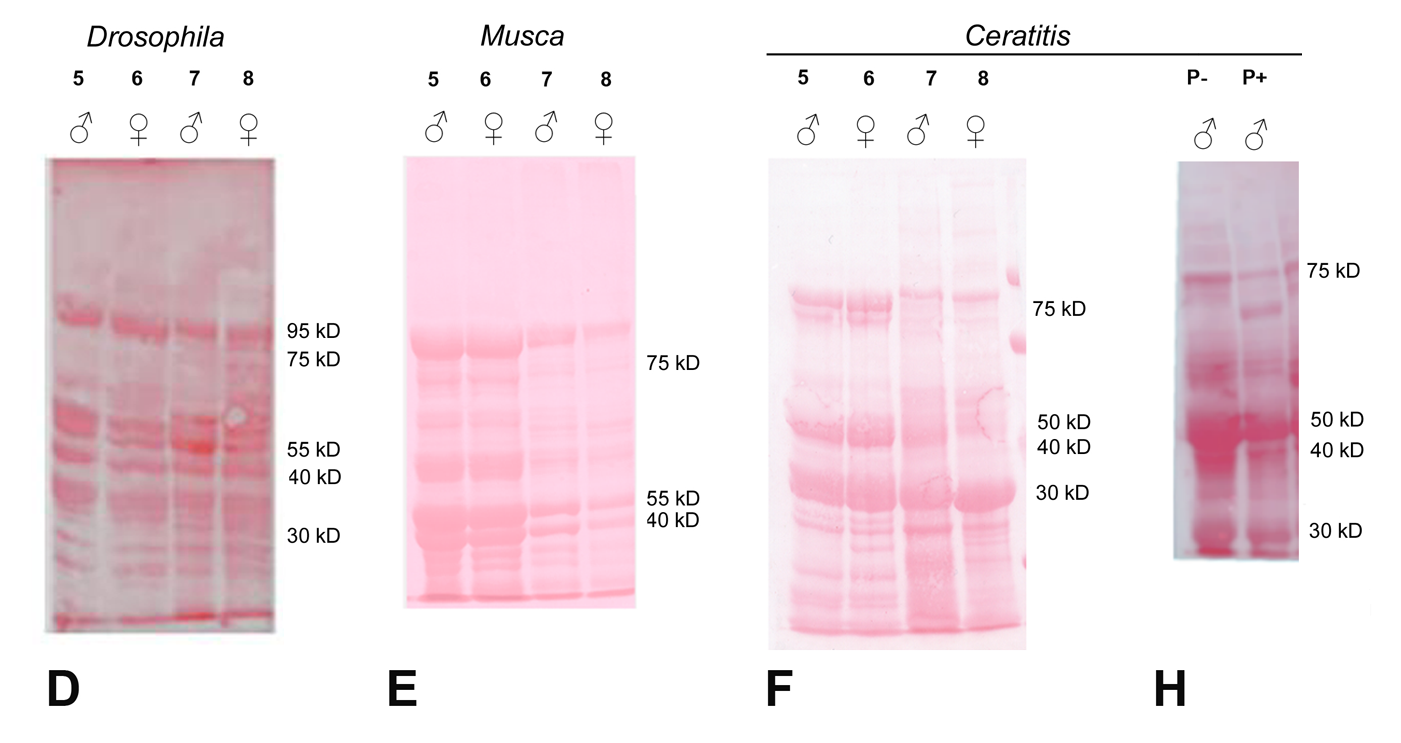

Supplement: Additional file 1 — Figure S1 Reversible red-ponceau staining of filters, used subsequently for mAb104 immuno-reactions. Figure 1: Ponceau staining of blots from SDS page electrophoresis of SR protein extracts of adult males and females of Drosophila (D), M. domestica (E) and C. capitata (F and G). 5) Mg++ supernatant of males; 6) Mg++ supernatant of females; 7) Mg++ pellets of males; 8) Mg++ pellets of females; P-) Mg++ pellets of C. capitata males without phosphatase treatment; P+) Mg++ pellets of C. capitata males with phosphatase treatment. [file 1471-2156-15-S2-S6-S1.tif]
